# Developmental Predictors of Suicidality in Schizophrenia: A Systematic Review

**DOI:** 10.3390/brainsci14100995

**Published:** 2024-09-30

**Authors:** Lindsay L. Benster, Noah Stapper, Katie Rodriguez, Hadley Daniels, Miguel Villodas, Cory R. Weissman, Zafiris J. Daskalakis, Lawrence G. Appelbaum

**Affiliations:** 1 Department of Psychiatry, UC San Diego, La Jolla, CA 92093, USA; llbenster@health.ucsd.edu (L.L.B.); nstapper@health.ucsd.edu (N.S.); klrodriguez@health.ucsd.edu (K.R.); hdaniels@health.ucsd.edu (H.D.); cweissman@health.ucsd.edu (C.R.W.); zdaskalakis@health.ucsd.edu (Z.J.D.); 2 Department of Clinical Psychology, San Diego State University, San Diego, CA 92182, USA; m1villodas@health.ucsd.edu

**Keywords:** schizophrenia, suicidal ideation, suicide attempts, risk factors, suicidality

## Abstract

**Background/Objectives**: Schizophrenia (SZ) is a severe psychiatric disorder characterized by a complex interplay of genetic, developmental, and environmental factors that significantly increase the risk of suicidal ideation (SI) and suicide attempts (SAs). This systematic review synthesizes current research on the developmental predictors of SI in individuals with SZ, aiming to delineate the multifactorial etiology of suicide within this population. **Methods**: A comprehensive search across Medline, PsycINFO, and EMBASE databases identified 23 eligible studies, emphasizing the varied methodological approaches and the global distribution of research efforts. **Results**: These studies demonstrate a robust association between early life adversities, particularly childhood trauma such as physical neglect, emotional abuse, and sexual abuse, and the increased prevalence of SI and SAs in SZ. This review also highlights the significant genetic factors associated with the development of suicidality in SZ, raising the possibility that polymorphisms in inflammation-related genes and neurodevelopmental abnormalities may influence susceptibility to SI. Notably, family history of psychiatric conditions may exacerbate the risk of SI through both hereditary and environmental mechanisms. Environmental factors, including socioeconomic status and social support, are also implicated, underscoring the role of broader socio-environmental conditions influencing outcomes. **Conclusions**: This review supports the integration of biopsychosocial models in understanding SI in SZ, advocating for interventions addressing the complex interplay of risk factors and the need for longitudinal studies to elucidate the dynamic interactions between risk factors over time. This comprehensive understanding is crucial for developing targeted preventive strategies and enhancing the clinical management of SZ, aiming to reduce suicidality in this vulnerable population.

## 1. Introduction

Schizophrenia (SZ) is a psychiatric condition marked by a profound disruption of thought processes and emotional responsiveness, posing a significant challenge for individuals, caregivers, and healthcare professionals [1]. SZ is associated with high economic and healthcare burden, with a 65% increase in prevalence, 37% increase in incidence, and 65% increase in disability-adjusted life years globally over the past three decades [2]. The symptomatology of SZ is diverse and debilitating, with positive symptoms such as hallucinations and delusions, negative symptoms including apathy and withdrawal, and cognitive impairments affecting memory and decision-making [1]. Given the developmental complexities confounding early psychotic symptoms, such as the potential for normal developmental experiences to be misconstrued, as well as trauma reactions and early substance use, SZ is typically not diagnosed prior to the age of 18 [3]. For the purpose of this review, developmental was defined as early life influences including genetic, demographic features, and environmental factors. Therefore, for this review, we only consider adult populations (age 18 and older) to accurately portray the disorder’s characteristics. SZ symptoms alone can make daily functioning and social integration profoundly difficult, leading it to being one of the world’s most disabling illnesses [4]. The complexity of this disease is not limited to its primary symptomatology, but also evident in frequent comorbidities, such as depression (up to 81%), anxiety disorders (~15% panic disorder alone), post-traumatic stress disorder (~29%), obsessive-compulsive disorder (~23%), and substance abuse disorders (~47%) [5,6]. These comorbidities not only exacerbate primary symptoms of SZ, but also introduce additional barriers to effective management and treatment, often leading to a more severe disease course.

Within this framework, suicidal ideation (SI) emerges as a major clinical concern, affecting a substantial proportion of those with SZ. Individuals with SZ often experience premature mortality, with up to 40% of this attributed to suicide and unnatural deaths [7,8]. Assessing prevalence rates of SI in SZ is far more challenging due to stigma, patient communication difficulties and limited insight, and fears of repercussions from disclosing SI, among others [9]. However, a recent meta-analysis estimates a 30 to 45% lifetime prevalence [10]. SI leads to increased disease burden, increases mortality, worsens quality of life, and interferes with treatment due to acuity. Furthermore, the experience of SI in SZ is not uniform, varying greatly in intensity and duration [1,11]. Evidence indicates that SI can be episodic or persistent and may fluctuate with changes in the severity of psychotic and psychopathological symptoms, highlighting the dynamic nature of suicide risk in this population [12]. Certain risk factors for SI in SZ have been suggested, including prior suicide attempts (SAs), family history of suicide, depressive symptoms, male gender, substance abuse, active hallucinations and delusions, and the degree of patient insight into their illness [7,13]. These risk factors are multifactorial and complex, involving an interplay of genetic vulnerabilities, neurobiological anomalies, and environmental influences that may act synergistically to precipitate suicidal thoughts and behaviors. Such factors may have a greater impact due to the cognitive and emotional challenges inherent in SZ, potentially hindering an individual’s ability to cope with stress, thereby increasing suicide risk. Despite the notable relationship between SI and SZ, a mechanistic understanding of this relationship is limited.

Developmental factors encompass a wide range of influences on SZ, marked by early life experiences and environmental stressors which shape individual’s susceptibility to psychopathologies and associated outcomes. The developmental hypothesis of SZ posits that the pathogenesis of the illness may begin long before the presentation of overt symptoms, during critical periods of brain development [14]. Adverse conditions during prenatal development, such as exposure to infection or malnutrition, have also been implicated in the etiology of SZ and may contribute to the later development of SI by altering brain structures and functions involved in stress and emotional regulation [15,16]. A large body of evidence supports the increased likelihood of developing SZ and experiencing SI later in life following adverse childhood experiences, such as trauma and neglect [17,18]. Additionally, neurodevelopmental abnormalities, including alterations in brain structure and function, have been implicated in both SZ and SI, highlighting the importance of understanding developmental trajectories from early life to adulthood as well as the disease onset and trajectory [19,20]. The cumulative effect of these predictors can shape an individual’s resilience or susceptibility to crisis points during the illness, particularly in the context of acute psychotic episodes or treatment transitions [21]. Despite decades of research, there remain substantial unknowns about the pathogenesis of SZ and the development of SI within this context. Therefore, elucidating the developmental predictors of SI within SZ is critical. This entails a comprehensive examination of an individual’s lifespan, from genetic predispositions and the influence of early life stressors to the onset of the initial psychotic episode and the enduring challenges posed by the disorder.

To our knowledge, no systematic reviews have previously been conducted assessing developmental predictors of SI in SZ. Therefore, this review aims to systematically explore the available literature to identify the developmental precursors that indicate elevated risk of SI among individuals with SZ. We aim to address the key developmental risk factors associated with SI in SZ and the specific genetic vulnerabilities that may interact with environmental stressors to influence the onset of SI in SZ. This understanding is essential for developing targeted interventions that can reduce the burden of suicide in this population and for informing future research directions.

## 2. Materials and Methods

Search Criteria: A systematic search of electronic databases including Medline, PsycINFO, and EMBASE was conducted between February 2024 and March 2024 to identify relevant studies published in English-language peer-reviewed journals. The search strategy utilized combinations of the following search terms: (“schizophrenia” OR “psychotic disorder” OR “psychosis”) AND (“SI” OR “suicidal thoughts” OR “suicidal behavior” OR “self-harm” OR “self-injury” OR “suicide risk”) AND (“developmental predictors” OR “risk factors” OR “early life factors” OR “early life stressors” OR “child* predictors” OR “adolescent predictors” OR “early adversity” OR “child* adversity” OR “adverse childhood experiences” OR “trauma*” OR “stressful life events” OR “victimization” OR “poly-victimization” OR “maltreat*” OR “abus*” OR “neglect*” OR “parental loss” OR “attachment” OR “social support” OR “family environment” OR “parent-child relationship” OR “peer relationships” OR “peer victimization” OR “socioeconomic status” OR “education level” OR “cognitive development” OR “emotional development” OR “neurodevelopment*” OR “brain development” OR “neurocognitive development” OR “genetic predisposition” OR “heritab*” OR “epigenetics” OR “gene-environment interaction”). Additionally, reference lists of relevant reviews and key studies were manually screened to identify additional eligible studies.

Inclusion and Exclusion Criteria: Studies were included if they focused on humans diagnosed with or meeting DSM-5 criteria for SZ aged 18 and above. Studies were excluded if they examined schizoaffective disorder, substance-induced or illness-induced psychotic disorders, clinical high-risk for psychosis, or depression with psychotic features. Existing evidence indicates there are potentially fundamental neurobiological differences distinguishing SZ from schizoaffective disorder; clinically this disorder is distinguished by the presence of mood features present occurring simultaneously with psychotic symptoms [22,23]. Furthermore, it is unclear whether SI in schizoaffective disorder is attributable entirely to mood features or other psychopathological experiences; thus, the focus of this review remains solely on SZ to better elucidate the relationship between SI and SZ. Eligible studies investigated SI, SA, suicidal thoughts, or suicidal behavior as outcome measures and examined various developmental predictors. Eligible studies encompassed empirical research with cross-sectional, cohort, case-control, or longitudinal designs. Additionally, only studies examining developmental predictors, risk factors, or precursors associated with SI in SZ were considered. Only full-text articles published in English-language peer-reviewed journals were included. In mixed samples, studies were only included if subgroup analyses were conducted focusing on those diagnosed with SZ. Publication types such as case reports, case series, editorials, reviews, commentaries, or conference abstracts were not considered. Studies with qualitative designs or those lacking primary data were also excluded. Furthermore, studies published in languages other than English were not included in the review.

Study Selection Process: Two independent reviewers (LB and NS) screened the titles and abstracts of identified studies to determine eligibility based on the inclusion and exclusion criteria. Full-text articles of potentially relevant studies were retrieved and assessed for eligibility. Discrepancies between reviewers were resolved through consensus or consultation with a third author if necessary. To ensure a consistent and accurate review process, the methodology was designed to maximize inter-rater consistency through structured review protocols and regular alignment meetings between the reviewers.

Data Extraction: Data from included studies were extracted using a standardized data extraction form. The extracted data included study characteristics (e.g., authors, year of publication, study design), participant characteristics (e.g., sample size, demographics), exposure variables (e.g., developmental predictors), outcome variables (e.g., measurement of SI), and key findings related to the association between developmental predictors and SI.

Quality Appraisal: The quality of included studies was assessed by two independent raters using the 11-item methodological checklist recommended by the Agency for Healthcare Research and Quality (AHRQ) for cross-sectional studies [24]. To enhance consistency and maintain the robustness of the review process, the assessment protocols were structured to promote uniform application across all studies, utilizing periodic discussions among reviewers to align on criteria and resolve any differences in the evaluation of study quality. Items were scored “1” only if they were answered as “Yes”; an item would be scored “0” when answered “No” or “Unclear”. The quality of the study was assessed as “low” when the score was 0–3; “moderate” when the score was 4–7; and “high” when the score was 8–11, consistent with similar reviews [25]. Studies classified as “low” quality were excluded from further analyses. Any discrepancy in assessment was resolved by a third author when necessary. Quality assessment criteria included sample representativeness, control of confounding variables, reliability, and validity of measurements (e.g., using standardized assessment tools such as the Structured Clinical Interview for DSM-5 Disorders), and appropriate statistical analysis methods (e.g., multivariate regression analysis).

Data Synthesis and Reporting: A narrative synthesis approach was utilized to summarize the findings of included studies. Themes and patterns related to developmental predictors of SI in SZ were identified and synthesized. The Preferred Reporting Items for Systematic Reviews and Meta-Analyses (PRISMA) guidelines were followed for reporting the systematic review process and findings.

## 3. Results

### 3.1. Descriptive Findings

#### 3.1.1. Study Selection

**Figure 1:**
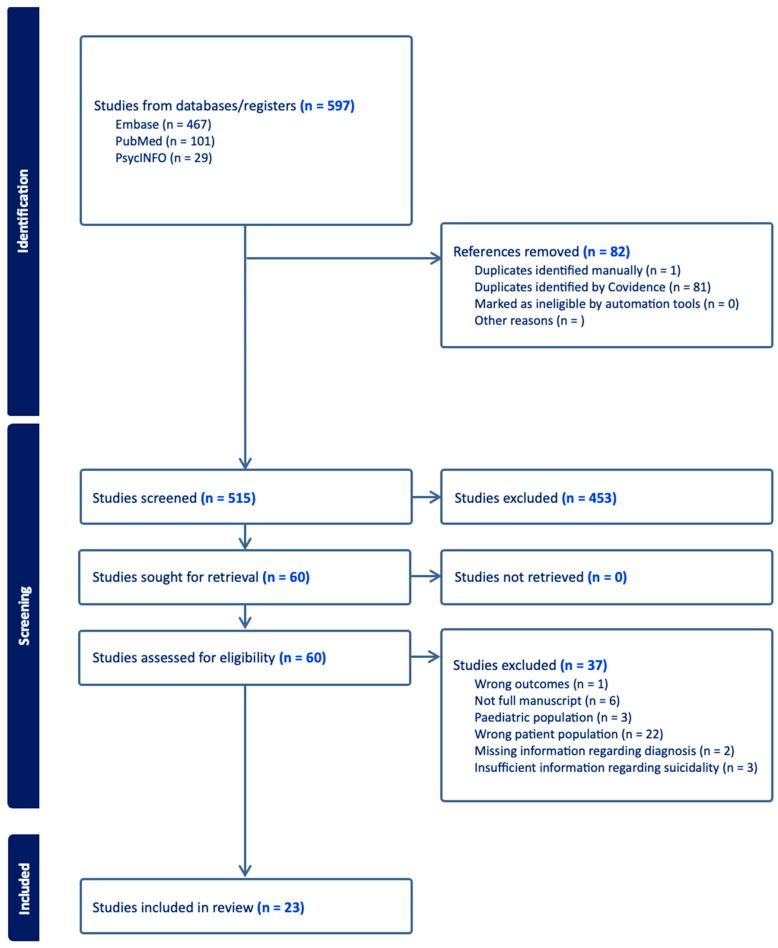
PRISMA Diagram of article selection process.

The selection process identified 23 eligible studies that met inclusion criteria for the current review. Selection procedure is illustrated in the PRISMA flow diagram (Figure 1). Initially, a pool of 597 records was identified using predefined search terms. Eighty-two references were removed for duplication, leaving 515 articles for title and abstract screening. Of these, sixty articles underwent full text examination. Twenty-three articles were determined to meet inclusion criteria. Studies were predominantly cross-sectional. These studies were published over a decade, from 2014 to 2024. The geographic dispersion of these studies was notable, with contributions from diverse cultural and healthcare contexts, ranging from metropolitan areas in Canada to rural districts in China, as well as in Europe and South America. The selected studies varied substantially in their methodological approaches, ranging from cross-sectional designs to retrospective analyses. Samples were predominantly inpatient or outpatient.

#### 3.1.2. Sample Characteristics

The reviewed studies encompass a broad array of sample sizes, ranging from small-scale investigations with 54 participants to large registries involving 668,836 individuals. The mean age of participants varied, with several studies reporting a mean in the early to mid-40s, although ages ranged from 18 to over 65 years, indicating inclusion of both young adults and older populations. The gender distribution was varied but often skewed towards a higher representation of male participants, with 18 out of 23 studies exhibiting a greater proportion of male participants, as illustrated in Table 1; the remaining five studies reported a majority of female participants or even distributions. Racial and ethnic backgrounds of participants were reported in some studies, with a number of studies highlighting a predominantly White European demographic, while others included more diverse populations with substantial proportions of Black, Hispanic, and other ethnic groups.

#### 3.1.3. Quality of Included Studies

Quality assessment revealed that most studies were methodologically sound, employing rigorous protocols and validated tools to measure SI with scores ranging from 6 to 10; thus, no studies were excluded for quality assurance purposes. All studies adhered to established diagnostic criteria for SZ using either the DSM or ICD, ensuring diagnostic consistency. Moreover, the methodological diversity among studies, ranging from surveys to cross-sectional and genome-wide association studies, provided a broad base to confirm the robustness of the identified predictors across different research paradigms.

#### 3.1.4. Assessment of Suicide

A variety of instruments were employed to measure SI and SA within this cohort. Three studies used the Columbia-Suicide Severity Rating Scale (C-SSRS), two studies utilized the Beck Scale for Suicide Ideation (BSSI), two studies employed the Mini International Neuropsychiatric Interview (MINI), one study applied the Calgary Depression Scale (CDS), one study reported using the Beck’s Suicide Intent Scale alongside nurses’ global assessment of suicide risk, one study measured with the Integrated Self-reported Suicidality Scale (ISST), one used the Suicide Behavior Questionnaire-Revised (SBQ-R), two studies incorporated the Self-rating Idea of Suicide Scale (SIOSS), and four studies identified death by suicide as their measure.

The CDS is specifically designed to assess depression in the context of SZ, and SI is measured by a singular item. The C-SSRS, nurses’ global assessment of suicide risk, and the BSSI are comprehensive tools that measure the severity and history of SI as well as the intensity of individuals’ desire to commit suicide; their frequent use across studies highlights their importance in clinical assessments of suicidality. The MINI offers a structured diagnostic interview that includes a module for assessing suicide risk. The ISST and the SIOSS are self-report measures that provide direct insights into the patients’ experiences and perceptions of their own suicidality. The SBQ-R and the Patient Health Questionnaire-9 (PHQ-9), a widely used self-report tool for assessing depression symptom severity, were employed in some studies for their utility in screening for suicide risk and depressive symptoms, respectively, with the latter assessing SI with a single-item and widely used in primary care settings.

#### 3.1.5. Assessment of Developmental Factors

The included studies encompassed an array of developmental predictors: socioeconomic and demographic factors (SES/demographic) were assessed in nine studies, indicating a recognition of the early social determinants of mental health outcomes. Childhood abuse, maltreatment, and early adversity were also prominently featured, with eleven studies exploring these factors, underscoring the critical impact of early life experiences on later mental health challenges. Genetics as a developmental predictor, assessed through either genome-wide association studies or exploration of specific genes of interest, was a focal point in six studies, reflecting an interest in the hereditary aspects of suicidal behaviors, while family history of SZ, SI, or psychiatric disorder influence was specifically evaluated in four studies. Environmental variables such as the first language being English and social influences were considered in four studies, suggesting an emerging interest in broader contextual factors.

### 3.2. Key Findings

Key findings for each of the 23 studies are summarized in Table 1.

**Table 1:** Risk Factors for Suicidality in SZ.

Study	Country	Study Design	Sample Size	Population Characteristics	Developmental Predictors	SI Metric	Findings
Acosta 2020 [26]	Spain	Cross-sectional	133	Outpatients Age: 46.7 (10.3) Gender: 69.2% M	SES	CDS	SA and SI groups exhibited higher SES versus non-suicidal group.
Aydın 2019 [27]	Turkey	Retrospective	223	Inpatients Age and Gender of suicide group: 41.0 ± 10.6 46% M	Family history of psychotic disorder, Lifetime traumatic event	SA	No group difference in family history. Significant difference in trauma type between SA and non-SA groups
Bani-Fatemi 2016 [28]	Canada	Cross-sectional	121	Inpatients Age: 45.3 ± 11.7 Gender: 65.5% M	Ethnicity, CT; Genetics/trauma interaction	C-SSRS; BSSI	White Europeans higher likelihood of SA. General abuse strongly linked to suicidal behavior. GWAS suggests interaction between SNPs and early CT but no genome-wide significance for SA in SZ
Bani-Fatemi 2018 [29]	Canada	Cross-sectional	123	Outpatients Age: 44.7 ± 12.3 Gender: 70% M	Genetics	C-SSRS; BSSI	Methylation at various gene regions differed between SZ patients with and without a history of SA
Bani-Fatemi 2020 [30]	Canada	Cross-sectional	107	Outpatients Age: 44.58 ± 9.14 Gender: 63.2% M	Genetics	CDS	Increased methylation of the *SMPD2* gene was observed among individuals with SI
Chang 2019 [31]	China	Retrospective	263	Inpatients Age: 64.11 ± 3.87 Gender: 19.1% M	SES/demographic; Other: Familry history of serious psychiatric problem; CT; Environmental factors	SA	SA in SZ linked to poor family relationships. No connection to other SES factors. Traumatic life events higher among SA patients.
Cheng 2022 [32]	China	Cross-sectional	91	Inpatients Age: 31.00 ± 7.78 Gender: 51.2% M	Childhood abuse/maltreatment; Early adversity	Beck’s suicide intent scale, nurses’ global assessment of suicide risk	Emotional neglect predicted suicide risk and SI, influenced by BMI linked to SMI. Relapse patients exhibit more CT and stress, uniquely associated with increased SI
Hu 2014 [33]	Canada	Case-control	234	Outpatients Age: 28.39 ± 9.1 Gender: 50% M	Genetics; SES/demographic	SA	*TH* gene TCAT(6) allele linked to 15-fold increased SA risk; TCAT(8) allele may be protective.
İngeç 2020 [34]	Turkey	Cross-sectional	200	Patients Age: 31.00 ± 7.78 Gender: 51.2% M	CT	MINI	All types of CT increase the risk of suicide
Kilicaslan 2017 [35]	Turkey	Cross-sectional	200	Inpatient and outpatient Age: 40.42 ± 11.20 Gender: 68.3% M	Childhood abuse/maltreatment; Early adversity; Family history of SI; Family history of SZ	MINI	CT predictive of SZ symptoms but not found significant predictor of SI
Lang 2020 [36]	China	Cross-sectional	1087	Inpatient Age: 47.8 ± 10.2 Gender: 81.9% M	Genetics	SA	No significant association was found between *TNF-alpha* gene polymorphisms and SZ or SA. The -1031C>T polymorphism linked to the age of first SA
Liu 2020 [37]	China	Cross-sectional	957	Inpatients Age: 47.8 ± 10.2 Gender: 81.8% M	Genetics	SA	The MTHFR polymorphism showed a weak correlation with SA in SZ. The Val/Val genotype was more prevalent among attempters
Lopez-Morinigo 2014 [38]	UK	Retrospective	54	Outpatient Age: 39.3 ± 11.9 Gender: 70.3% M	Demographic info	Death by suicide	SZ suicide deaths were often younger, of Black origin, English as a first language, and more socially deprived compared to non-SZ suicide deaths
Lyu 2021 [39]	China	Psychological Autopsy (PA)	38	Survey Age: 29.03 ± 5.592 Gender: 39.5% M	SES; Environmental factors	Death by suicide	No significant differences in education, residence, marital status, living alone, or family size between SZ and non-SZ suicide groups, but significant differences in age and gender, with female patients more likely to die by suicide. Lower levels of social support in SA groups
Mohammadzadeh 2019 [40]	Iran	Cross-sectional	82	Inpatient Age: 34.78 ± 9.10 Gender: 41.5% M	CT	BDI; BSSI	High levels of CT linked to more severe SI. Genderual abuse was a unique predictor of lifetime SA, while physical neglect and depression unique predictors of current SI
Nath 2021 [41]	India	Cross-sectional	140	Inpatients Age: 31.17 ± 8.5 54.3% M	Family history, SES	ISST	SI was significantly associated with a family history of psychiatric illness (especially SZ), and suicide. No relationship with SES factors
Olfson 2021 [42]	United States	Retrospective	668,836	Medicare beneficiaries Gender: 52.5%M	Resources/medicare/SES	Death by suicide	Medicare patients with SZ had a 4.5-fold increased risk of suicide, with higher risk for men, Whites.
Prokopez 2020 [43]	Argentina	Cross-sectional	100	Inpatients Age: 45.82 (±12.68) Gender: 50% M	CT	C-SSRS	Women with ≥5 ACEs had higher death ideation, SA frequency, and SA median numbers. Emotional abuse most common ACE in SZ.
Taktak 2023 [44]	Turkey	Cross-sectional	222	Outpatients Age: 45.92 ± 12.31 Gender: 70.27% M	SES	SBQ-R	Higher SI associated with high school education and lower income
Xia 2018 [45]	China	Retrospective	223	Inpatient Age: 48.1 9.6 years) Gender: 84% M	SES/CT	SA	Marital status, gender, employment, education, family history of psychiatric illness, cohabitation, and living situation were not linked to SA. Traumatic life event history was higher in those with past SA.
Xie 2018 [46]	China	Cross-sectional	216	Outpatients Age: 27.78 ± 8.13 Gender: 55.5% M	CT	SIOSS	SI positively correlated with CT severity and variety; inversely related to social support
Yu 2024 [47]	China	Cross-sectional	281	Inpatients Gender: 58.7% M	CT/environmental factors	PHQ-9; SIOSS	CT and psychological resilience influenced the onset of SI in SZ
Zhang 2021 [48]	Canada	Cross-sectional	83	Inpatients Age: 38.39 ± 10.23 Gender: 54% M	CT/physical neglect	BSSI	SI was related to insomnia and CT, with physical neglect identified as an independent risk factor

SES = Socioeconomic Status; CDS = Calgary Depression Scale; SA = Suicide Attempt; SI = Suicidal Ideation; C-SSRS = Columbia-Suicide Severity Rating Scale; BSSI = Beck Scale for Suicidal Ideation; GWAS = Genome-Wide Association Study; SNP = Single Nucleotide Polymorphism; SZ = Schizophrenia; SMPD2 = Sphingomyelin Phosphodiesterase 2; CT = Childhood Trauma; BMI = Body Mass Index; MINI = Mini International Neuropsychiatric Interview; TH = Tyrosine Hydroxylase; ISST = Integrated Self-reported Suicidality Scale; BDI = Beck Depression Inventory; ACE = Adverse Childhood Experiences; SBQ-R = Suicidal Behaviors Questionnaire-Revised; SIOSS = Suicidal Ideation of Self Scale; PHQ-9 = Patient Health Questionnaire-9.

#### 3.2.1. Sociodemographic Factors

Evidence for gender-related differences was mixed. Lyu et al. [39] observed females with SZ were more prone to suicide. Contrarily, Olfson et al. [42] found that suicide risk among patients with SZ was higher for men than for women. However, other research, such as that by Xia et al. [45] and Chang et al. [31], found no significant difference in SA rates between genders, indicating that while gender may play a role in SI, it is not a consistent predictor across different cultural or geographic contexts.

Studies have further highlighted age as a pivotal factor in SI among patients with SZ. Notably, the mean age of participants with SI was often found to be lower than those without SI, suggesting that younger patients with SZ might bear a higher risk. Olfson [42], reported that suicide risk was significantly higher in younger adults with SZ and tended to decline with each passing decade, and Lopez-Morinigo [38] revealed that individuals with SZ who died by suicide were typically younger. Taktak (2023) [44] also found that higher SBQ scores were associated with younger age, reinforcing the notion that younger individuals with SZ face an elevated risk of SI. To the contrary, Lyu et al. [39] reported that older females with SZ in rural China were more likely to exhibit suicidal behavior compared to younger individuals without SZ, indicating that age-related risks may differ across genders and cultural settings. Lang et al. [36] highlighted a significant positive correlation between the age of SZ onset and age of first SA.

Socioeconomic status (SES) appears to be potentially relevant to the SI risk in SZ, but the findings are mixed. Taktak (2023) [44] reported that lower income and degree of education were associated with higher SBQ scores, indicating a potential link between economic hardship and SI. However, Chang et al. [31] and Lyu et al. [39] observed that factors including education level, features of residence, and family size did not significantly influence SA rates. Acosta et al. [26] found SA and SI groups exhibited higher SES than non-suicidal SZ groups. Olfson et al. (2021) [42] suggested that other SES factors, such as being a Medicare patient, increased the prevalence of suicide 4.5-fold among individuals with SZ compared to the general population. This suggests that while SES factors such as income and educational attainment may contribute to SI, their influence is complex and potentially mediated by other variables.

Ethnicity has been identified as a factor of interest in suicide risk among patients with SZ. Olfson [42] found significant differences in suicide risk among ethnic groups, with White patients exhibiting a higher risk compared to Black and Hispanic patients. Bani-Fatemi [28] corroborated this, finding White Europeans had a higher likelihood of SA than non-white individuals. In contrast, Lopez-Morinigo et al. [38] revealed serious SI was more prominent in Black individuals and those with English as a first language, suggesting that ethnicity-related factors may differentially affect suicide risk and require further exploration in various cultural contexts.

Across several studies, younger age and White Caucasian ethnicity were significant predictors of SI in patients with SZ. Other sociodemographic variables such gender and SES have been shown to interact in a complex manner in influencing suicidal behaviors among SZ patients, but without clear directionality. Nath et al. [41] and Xia et al. [45] did not find a significant correlation between age, gender, religion, educational level, and SI.

#### 3.2.2. Genetic Associations

The genetic underpinnings of SI have been underscored by findings linking SI with polymorphisms in inflammation-related genes such as C-reactive protein (CRP), highlighting the involvement of biological pathways. These have been less explored within SZ. Lang et al. [36] examined the tumor necrosis factor (*TNF)-alpha* polymorphism, known for its role in inflammatory processes, and discovered its association with the timing of first SA in chronic SZ patients. Their findings indicated no direct connection between the *-308G>A* and *-1031C>T* polymorphisms of the *TNF-alpha* gene with either SCZ or SAs. However, they identified a novel association where the *-1031C>T* polymorphism was related to the age at which patients first attempted suicide, with patients carrying the C allele attempting SA later than TT genotype carriers. This finding points to a potential genetic marker that could help predict the onset of SAs in this group. Similarly, Liu et al. [37] investigated the *MTHFR Ala222Val* polymorphism and its link to SAs, finding only a weak correlation. The *MTHFR* gene (methylenetetrahydrofolate reductase) plays a crucial role in the body’s metabolism of folate and regulation of homocysteine levels—an amino acid associated with various cardiovascular and neurological conditions. Elevated homocysteine levels have been linked to various psychiatric disorders, including depression and SZ. Their research revealed no substantial difference in the prevalence of this polymorphism between SZ patients and healthy controls, aligning with previous findings, which suggest that the *Ala222Val* polymorphism does not enhance the risk for SZ. This study highlighted a less frequent occurrence of the *Ala/Val* genotype and a more common presence of the *Val/Val* genotype in SZ patients who have attempted suicide compared to those who have not, suggesting the *Val/Val* genotype might be associated with an increased risk of suicidal behavior. Furthermore, the *MTHFR Ala222Val* polymorphism did not correlate with general psychopathology severity, as indicated by PANSS scores, suggesting that its impact might be specific to suicidal tendencies.

Hu et al. [33] pinpointed specific variants of the *tyrosine hydroxylase (TH)* gene, a rate-limiting enzyme of catecholamine synthesis, playing a critical role in the production of dopamine, norepinephrine, and epinephrine, associated with SA in SZ patients. Specifically, they reported the *TCAT(6)* allele of the *TH* gene might be an independent risk factor for SAs, with its effect persisting beyond the influence of clinical variables, such as hospitalizations. In addition to the *TCAT(6)* allele, Hu et al. [33] identified a haplotype within the *TH* gene that could independently predict SAs, although they cautioned that due to its rarity, further validation in larger cohorts is required. They also observed a potential protective effect of the *TCAT(8)* allele against SAs.

The series of studies by Bani-Fatemi et al. (2016, 2018, and 2020) [28,29,30] explored various genetic influences on suicidal behavior. Their 2016 study underscored the impact of sexual abuse as a significant factor, suggesting it interacts with specific SNPs to influence suicidal behavior. The 2018 study focused on the role of epigenetic variation, finding DNA methylation at various gene regions differed between SZ patients with and without a history of SA. Specifically, hypomethylation at site *cg19647197* within the *CCDC53* gene was noted in SZ attempters. The 2020 study examined the relationship between genome-wide methylation status and SI, finding the *SMPD2* gene was not linked to SI. The SLC20A1 gene showed an association with SI, indicated by differences in methylation patterns with increased methylation of the *SMPD2* gene in individuals with SI.

#### 3.2.3. Childhood Adversity/Trauma

Early life adversity, including childhood trauma and maltreatment, has been identified as a significant factor influencing the prevalence and severity of SI and SA in individuals with SZ. Yu et al. [47], İngeç and Kilicaslan [34], and Cheng et al. [32] observed that all forms of abuse increased the risk of suicide and SI. Cheng et al. (2022) [32] specifically addressed this in Chinese patients with SZ, highlighting that experiences of physical abuse, sexual abuse, and especially emotional neglect during childhood have profound implications for the onset of psychosis. They posited emotional neglect as a significant risk factor for increased suicide risk and behavior. Their findings suggest that childhood maltreatment not only has a direct impact on the risk of suicide but also influences the severity of the disorder’s symptomatology. The study by Bani-Fatemi et al. (2016) [28] further expanded on this, indicating that sexual abuse was a robust factor contributing to the likelihood of suicidal behavior, even after controlling for depression severity. In line with the existing literature on the exacerbating effects of trauma, Aydin et al. [27], Chang et al. (2019) [31], and Xia (2018) [45] found an elevated occurrence of traumatic life events in SZ patients with a history of SAs. Aydin et al. (2019) reported the following breakdown of traumatic life events in individuals with a history of SA: traumatic life events overall in 38.6%, emotional trauma in 17.9%, physical trauma in 16.1%, sexual trauma in 4.5%, and overall, a significant difference in trauma type between SA and non-SA groups [27].

A study by Mohammadzadeh et al. [40] found higher levels of childhood trauma were associated with more severe SI and symptoms, and specific types of trauma, notably sexual abuse, and physical neglect, were uniquely predictive of previous SAs and current SI, respectively. Importantly, after controlling for depression, only emotional abuse and physical neglect remained significantly associated with current SI. However, in more detailed analyses, sexual abuse emerged as a significant predictor of previous SA. Prokopez [43] noted higher frequencies in patients with a history of multiple ACEs. Specifically, women with ≥5 ACEs had higher death ideation, SA frequency, and SA median numbers. Emotional abuse was found to be the most common ACE in SZ. Xie et al. (2018) found SI positively correlated with childhood trauma, with correlations between severity and variety [46]. The study by Zhang et al. (2021) [48] confirmed that patients with SZ experiencing SI report higher rates of childhood trauma, particularly physical neglect, compared to those without such ideation. Only one study [35] reported no significant link between childhood trauma and SI. However, this study did report a correlation between childhood physical abuse and SZ symptomology. Overall, 10 out of 10 studies found correlations between childhood trauma and suicidality.

#### 3.2.4. Family History

Two studies, both Xia et al. (2018) and Aydın and colleagues [27], reported no significant difference between groups with and without a history of SA in terms of family history of psychiatric illness [45]. However, contrasting findings were presented by Nath and colleagues (2021) [41] and Kilicaslan and colleagues [35], who reported that a family history of SZ and suicide, respectively, were significantly associated with SI.

#### 3.2.5. Environmental Factors

Environmental factors constitute an array of influences including family dynamics, parental mental health, and the broader socio-cultural context in which individuals are raised and live. These factors can have a substantial impact on the psychiatric outcomes of SZ patients. Lyu et al. (2021) [39] examined differences in suicide cases between individuals with and without SZ, finding that while SES did not vary significantly, there were marked differences in social support received. Specifically, the SZ group who died by suicide had notably lower levels of social support compared to those without the disorder. Cheng et al. (2022) [32] found that the quality of the environment post-childhood maltreatment may either exacerbate or mitigate the risks associated with early life adversity. In other words, a supportive and positive environment following early adverse experiences may lessen the long-term negative outcomes associated with such trauma. Conversely, a negative or unsupportive environment post-childhood maltreatment increased the risks. The study by Zhang et al. (2021) [48] highlighted that while childhood trauma was significant, factors such as poor social functioning, social isolation, and withdrawal, can exacerbate the risk of SI in SZ patients. Chang et al. (2019) [31] also discussed the importance of family relationships, finding that poor family bonds were significantly associated with lifetime SAs. Similarly, Lopez-Morinigo compared individuals with and without SZ who died by suicide and reported SZ patients were more socially deprived compared to non-SZ. Yu et al. [47] reported resilience (defined as psychological resources, internal and personal assets, appraisals, and/or qualities that prevent suicidal thoughts and behaviors and can protect individuals from the impact of adverse events and their aftermath) as a protective factor, potentially mitigating the impact of negative experiences and stressors commonly encountered by SZ patients.

## 4. Discussion

This systematic review critically examined the developmental predictors of suicidality among individuals with SZ. Developmental was defined as early life influences including genetic, demographic features, and environmental factors. The primary objective was to elucidate how early-life experiences, genetic predispositions, and environmental factors contribute to suicidality in SZ, an issue of growing concern given the substantial morbidity and premature mortality associated with the disorder. Key aims included identifying specific developmental risk factors, understanding the interaction between genetic vulnerabilities and environmental stressors, and exploring potential interventions to mitigate suicide risk. The main findings indicated that early-life adversities, such as childhood trauma, including physical neglect, emotional abuse, and sexual abuse, significantly increase the risk of SI and SA in individuals with SZ. A broad overview of the consistency of these findings across each variable is summarized in Supplementary Materials, Table S1. Genetic factors, including polymorphisms in inflammation-related genes and neurodevelopmental abnormalities, also contribute to suicidality, potentially interacting with environmental stressors to heighten risk. Additionally, a family history of psychiatric conditions was identified as a potential risk factor, suggesting both hereditary and environmental influences. Socioeconomic status and lack of social support were further implicated as environmental risk factors that exacerbate the likelihood of suicidality. The review highlights the complex interplay of biopsychosocial factors in driving SI in SZ, underscoring the need for targeted interventions that address these multifaceted risks and for longitudinal studies to further clarify the dynamic interactions between these predictors over time.

The studies suggest that sociodemographic factors, such as gender, age, and marital status, may not directly impact the prevalence of suicidal behaviors in SZ, but could influence how other factors affect outcomes. Given the inconsistent findings, SES likely interacts with a multitude of other individual and contextual factors, including the vocational challenges faced by SZ patients. This underscores the need for context-sensitive interventions that consider how demographic elements interact with clinical and environmental factors. The mixed findings across studies accentuate the necessity for localized strategies tailored to specific community and cultural dynamics, potentially guiding more effective prevention efforts by addressing the unique needs of individual patients. Convergent findings across studies suggest that younger age and White ethnicity are significant predictors of suicidality in SZ, though these relationships may vary based on cultural and contextual factors. The studies examining English speakers appears to not be directly about the language itself, but rather it represents a proxy for broader socio-cultural and environmental factors. For instance, individuals who do not speak the dominant language may face significant social isolation, reduced access to mental health services, and communication barriers, all of which are known to exacerbate psychological distress and elevate the risk of suicidality. Overall, the research indicates that the relationships between sociodemographic factors and suicidality in SZ are not straightforward and require consideration of the broader psychosocial context.

The research indicates that early life adversity and childhood trauma are critical risk factors. This was found consistently across the included studies with one exception, underscoring the impact of childhood experiences like physical neglect, emotional abuse, and sexual abuse. These findings reinforce the stress-diathesis model, suggesting that inherent vulnerabilities combined with traumatic experiences heighten suicide risk within this population. This insight is critical for developing targeted interventions that address the unique developmental trajectories and resilience factors of individuals exposed to early adversity. Recognizing these needs can improve the timing and customization of therapeutic approaches, potentially reducing suicide rates in SZ populations. This understanding invites a broader integration of trauma-informed care within mental health services, advocating for early intervention strategies that preemptively address the repercussions of childhood trauma before they manifest into severe psychopathological conditions in adulthood.

Depression and the severity of psychotic symptoms have emerged as significant contributors to suicidality in schizophrenia, warranting careful consideration. In adult populations, comorbid depression, which is prevalent in up to 32% of individuals with schizophrenia, substantially increases suicide risk by compounding the emotional and cognitive burdens faced by patients [49]. Depression exacerbates feelings of hopelessness and despair, both of which are key drivers of suicidal ideation and behaviors [50]. Moreover, the severity of psychotic symptoms, including treatment-resistant cases and substance use that fuels psychosis, further intensifies the risk of suicide. Treatment-resistant schizophrenia often leads to chronic distress, diminished quality of life, and a sense of entrapment, which are critical factors that precipitate suicidality [51]. Understanding the synergistic impact of these factors with developmental predictors is crucial for developing comprehensive prevention strategies.

The discovery of genetic associations highlights the potential for pharmacogenomics to personalize psychiatric care based on genetic profiles. While the current application of genetic findings is limited, their integration into theoretical frameworks like the diathesis-stress model can clarify how genetic predispositions interact with environmental stressors to modulate risk. For example, polymorphisms in inflammation-related genes such as CRP and TNF-alpha suggest that inflammatory processes could be central to these interactions, possibly affecting an individual’s response to psychiatric stress and trauma. The potential protective effect of the TCAT(8) allele against SAs aligns with previous research on bipolar disorder, thereby hinting at possible common genetic factors underlying suicidality across both these psychopathologies, rather than a unique characteristic of SZ. Furthermore, genes like MTHFR, which is involved in folate metabolism and consequently affects homocysteine levels, could link metabolic states to psychiatric conditions and suicidality, offering new avenues for intervention. While no single genetic marker has emerged as a definitive predictor, the interaction between multiple genetic factors and early life experiences suggests a cumulative risk model.

The significance of family history in our findings resonates with both genetic theories and attachment theories, the latter of which emphasizes the impact of early relational experiences on the development of coping mechanisms. Clinically, this may continue to encourage the integration of family dynamics into treatment plans, with an emphasis on creating secure attachment experiences and addressing intergenerational patterns of psychiatric disorders. Family history, especially of psychiatric illnesses, has been consistently shown to compound the risk, indicating both a hereditary component and the potential impact of growing up in an environment shaped by mental illness. Here, there were mixed findings regarding family history. However, the existing literature strongly suggests a positive correlation [52], and due to the minimal invasiveness and cost associated with collecting a family history, it is a promising risk factor worth assessing. Further, it is evident that individuals with SZ with a family history of psychiatric disorders are at a heightened risk of suicidality.

Findings presented here resonate with patterns observed in other psychiatric disorders, such as mood and schizoaffective disorders, where childhood trauma impacts disease severity and suicidality [53]. ACEs are well-documented risk factors for a range of psychiatric disorders, including depression, anxiety, and substance use disorders [54]. However, their impact may be particularly pronounced in schizophrenia due to the disorder’s unique neurodevelopmental trajectory [55]. In schizophrenia, ACEs not only exacerbate the severity of psychotic symptoms but may also contribute to earlier onset and poorer long-term outcomes [43]. The combination of genetic vulnerability and environmental stress from ACEs may trigger neurodevelopmental disruptions, leading to a higher susceptibility to suicidality in individuals with schizophrenia compared to other mental health disorders. Similarly, genetic factors implicated in SZ, like the *MTHFR* and *TH* gene variations, are also relevant in other psychopathologies, suggesting common neurobiological pathways that increase susceptibility to psychiatric conditions and suicidality. Moreover, the complex interplay between sociodemographic factors and suicidality noted in SZ mirrors challenges faced in managing other mental health disorders, where factors such as age, gender, and SES significantly influence disease outcomes and treatment responses. Further, it is imperative to address the complexity and interconnectedness of variables under review in this manuscript. For instance, factors such as poor early-life social support and low SES may not only reflect immediate environmental stressors, but can also serve as indirect indicators of a familial predisposition to mental health challenges. For instance, parents struggling with their own mental health burdens may be less capable of providing adequate social support or achieving higher SES, thereby transmitting both genetic vulnerabilities and environmental stressors to their children. This points to a shared psychosocial context that could benefit from holistic, integrated care approaches.

In synthesizing these factors, it is clear that an integrated approach to risk assessment for suicide in SZ must consider the cumulative and synergistic effects of early life adversity and specifically maltreatment, genetic predisposition, family history, and environmental factors. This comprehensive understanding can inform targeted interventions and support systems to mitigate the multifactorial risks associated with suicide in SZ.

### 4.1. Limitations

There review has several limitations. Firstly, within the literature itself, there was notable diversity in the operationalization of SI and the timeframes in which it was assessed. The lack of standardization across studies and varied definitions complicates the ability to draw broad conclusions. Differences in suicide measurement methods also poses challenges in comparing findings. Additionally, the scope of studies reviewed often limited the exploration of race and ethnicity as modifiers of SI risk in schizophrenia, restricting the generalizability of findings across diverse populations. The focus of this review was intentionally narrowed to focus on environmental, genetic, and demographic factors. Consequently, factors such as neurocognitive development, brain imaging, and physiological markers, though important, were outside the scope of this review. Another limitation is the predominance of cross-sectional designs, limiting the ability to ascertain causality and assess longitudinal impact of developmental risk factors. This underscores the need for more rigorous and replicable research methodologies to enhance the reliability of findings in schizophrenia and suicidality research. The methodological limitations of this systematic review also warrant careful consideration. Despite efforts to minimize subjectivity, the inclusion and exclusion criteria and data extraction processes were based on the reviewer judgment, potentially introducing bias. Furthermore, the quality assessment of the included studies revealed variability, indicating that not all studies met the highest methodological standards.

### 4.2. Implications

The development of standardized, sensitive assessment tools is crucial for accurately predicting suicidal tendencies, enabling more targeted suicide prevention interventions for SZ. Policy-level initiatives should focus on creating environments that reduce risk factors like social isolation and stigma. This review underscores the necessity for a robust research agenda that includes longitudinal studies and replication to clarify causal relationships and establish effective, broadly applicable interventions. Furthermore, enhancing healthcare provider education on the latest diagnostic and therapeutic approaches for managing SI in SZ is essential for improving patient outcomes. Future directions in research might benefit from longitudinal designs that can track the progression of risk factors over time, possibly revealing dynamic interactions that cross-sectional designs cannot capture. Additionally, prospective studies examining early intervention for high-risk groups based on identified risk factors as presented here would be beneficial. Moreover, addressing the limited generalizability of studies by including diverse populations can shed light on cultural and socioeconomic variations in risk factors and their manifestations.

## 5. Conclusions

This systematic review highlights the intricate web of developmental factors that contribute to suicidality in individuals with SZ. The findings underscore the pivotal role of early life adversities, particularly various forms of childhood trauma, and genetic predispositions that interact with environmental stressors, magnifying the risk of suicidality. Moreover, the impact of familial psychiatric history and socio-environmental conditions, such as SES and social support, elucidates the complex interactions that exacerbate this risk. The review calls for a multidimensional approach to understanding and managing suicidality in schizophrenia, emphasizing the need for integrated biopsychosocial interventions. Future research should pursue longitudinal studies to capture the dynamic nature of these risk factors and enhance the generalizability of findings across diverse cultural and socioeconomic contexts, ultimately aiding in the development of more effective preventive and therapeutic strategies for this vulnerable population.

## Data Availability

The data presented in this review are openly available.

## Supplementary Materials

The following supporting information can be downloaded at: https://www.mdpi.com/article/10.3390/brainsci14100995/s1, Table S1: Consistency of Key Findings.
