# Supplementary material for: Developmental Predictors of Suicidality in Schizophrenia: A Systematic Review
Source: Brain Sci. 2024 Sep 30;14(10):995. doi: 10.3390/brainsci14100995 (PMC11506348; doi:10.3390/brainsci14100995)
Supplement: Supplementary file 1 [file brainsci-14-00995-s001.zip › brainsci-3233776-supplementary.pdf]

brain sciences

MDPI

**Supplemental Materials**

**Table S1.**

| Variable | Number of Studies Examined | Number of Consistent Findings | Directionality/Details of Consistent Findings |
|---|---|---|---|
| **Socioeconomic Status (SES)** | 9 | 3 | Higher SES linked with lower risk of suicidality in 3 studies |
| **Childhood Trauma** | 11 | 10 | Consistent findings showing higher risk of suicidality with childhood trauma in 10 studies |
| **Genetics** | 6 | 5 | Significant genetic associations found in 5 studies, mainly focusing on gene polymorphisms |
| **Family History** | 4 | 2 | Family history of psychiatric illness showed inconsistent results; 2 studies found a positive correlation |
| **Environmental Factors** | 4 | 3 | Lower social support and adverse family environment consistently associated with higher suicidality in 3 studies |
